# Promoting Active Transport in Older Adolescents Before They Obtain Their Driving Licence: A Matched Control Intervention Study

**DOI:** 10.1371/journal.pone.0168594

**Published:** 2016-12-29

**Authors:** Hannah Verhoeven, Dorien Simons, Jelle Van Cauwenberg, Delfien Van Dyck, Corneel Vandelanotte, Bas de Geus, Ilse De Bourdeaudhuij, Peter Clarys, Benedicte Deforche

**Affiliations:** 1 Department of Public Health, Faculty of Medicine and Health Sciences, Ghent University, Ghent, Belgium; 2 Physical Activity, Nutrition and Health Research Unit, Faculty of Physical Education and Physical Therapy, Vrije Universiteit Brussel, Brussels, Belgium; 3 Research Foundation - Flanders (FWO), Brussels, Belgium; 4 Department of Movement and Sport Sciences, Faculty of Medicine and Health Sciences, Ghent University, Ghent, Belgium; 5 Physical Activity Research Group, School for Human, Health and Social Science, Central Queensland University, Rockhampton, Australia; 6 Human Physiology Research Group, Faculty of Physical Education and Physical Therapy, Vrije Universiteit Brussel, Brussels, Belgium; University of Edinburgh, UNITED KINGDOM

## Abstract

**Background:**

Active transport has great potential to increase physical activity in older adolescents (17–18 years). Therefore, a theory- and evidence-based intervention was developed aiming to promote active transport among older adolescents. The intervention aimed to influence psychosocial factors of active transport since this is the first step in order to achieve a change in behaviour. The present study aimed to examine the effect of the intervention on the following psychosocial factors: intention to use active transport after obtaining a driving licence, perceived benefits, perceived barriers, subjective norm, self-efficacy, habit and awareness towards active transport.

**Methods:**

A matched control three-arm study was conducted and consisted of a pre-test post-test design with intervention and control schools in Flanders (northern part of Belgium). A lesson promoting active transport was implemented as the last lesson in the course ‘Driving Licence at School’ in intervention schools (intervention group 1). Individuals in intervention group 2 received this active transport lesson and, in addition, they were asked to become a member of a Facebook group on active transport. Individuals in the control group only attended the regular course ‘Driving Licence at School’. Participants completed a questionnaire assessing socio-demographics and psychosocial variables at baseline, post (after one week) and follow-up (after eight weeks). To assess intervention effects, multilevel linear mixed models analyses were performed.

**Results:**

A sample of 441 older adolescents (56.8% female; 17.4 (0.7) years) was analysed. For awareness regarding the existence of car sharing schemes, a significant increase in awareness from baseline to post measurement was found within intervention group 1 (p = 0.001) and intervention group 2 (p = 0.030) compared to the control group in which no change was found. In addition, a significant increase in awareness from baseline to follow-up measurement was found within intervention group 1 (p = 0.043) compared to a decrease in awareness from baseline to follow-up measurement within the control group.

**Conclusions:**

Overall, the intervention was not effective to increase psychosocial correlates of active transport. Future intervention studies should search for alternative strategies to motivate and involve this hard to reach target group.

## Introduction

Active transport (e.g. walking and cycling) has great potential to increase physical activity in adolescents and young adults since it can be easily integrated into the daily routine [1–3]. It offers health benefits such as the prevention of overweight or obesity [4, 5], higher levels of cardiovascular fitness [6, 7] and a better cognitive performance [8]. Increasing active transport may also be beneficial to the environment and public health, as an increase in active transport may reduce traffic congestion and CO_2_ emissions [9]. Despite numerous advantages of active transport, a steep decline occurs during adolescence and continues when entering adulthood [1]. A study in 10 European cities showed that cycling for transport decreased from 30 to 25 minutes per day between 12.5–13.9 and 14–14.9 years, and from 25 to 20 minutes per day between 14–14.9 and 15–17.4 years [1]. Furthermore, a study among Danish adolescents showed that active transport accounted for around 20% of daily minutes of moderate-to-vigorous physical activity (MVPA) [10], whereas a study in Belgium found that adults spent 57% of their daily minutes of MVPA in active transport [11] because of lower total physical activity levels.

Once adolescents reach driving age, their behaviour changes dramatically [12]. Acquiring a driving licence has a substantial negative effect on adolescents’ (16–18 years) active transport with a 40% decline in the average number of walk trips [12]. A qualitative study among adolescents in New Zealand also indicated that driving became the preferred transport mode to school once they obtained a car driving licence [13]. In most European countries, adolescents have the possibility to obtain a regular car driving licence from the age of 18. In 2013, 49.3% of 18–24 year olds in Flanders (northern part of Belgium) possessed a driving licence [14]. Once habits toward a particular behaviour are formed, they are difficult to change [15]. When travel behaviour has become habitual, a particular travel goal automatically activates a travel mode in memory since people fail to suppress the habitual travel mode option in favour of alternative travel modes [15]. Therefore, it might be important to promote active transport at the age of 17–18 years (older adolescence), which may represent a crucial period for intervening before habitual car driving patterns get established.

A variety of interventions to promote walking and cycling as a mode of transport have been introduced using a range of methods in multiple settings (such as schools, workplaces, communities and households) among various age groups [16–22]. Mixed results were found across these studies. Several intervention studies targeted (working) adults [16, 18, 20, 21] although their transport behaviour had often already evolved into a habitual behaviour which is difficult to change. Few studies have targeted youth and those who did mainly targeted primary school children and younger adolescents (12–16 years old) [17, 19, 22]. To the best of our knowledge, no intervention studies focused on older adolescents although this is an important age group just before a critical transition regarding transport behaviour.

In order to achieve changes in behaviour, it is an important strategy for intervention studies to target the correlates of that behaviour. According to Epton et al. [23], the Theory of Planned Behaviour provides a strong theoretical framework for developing interventions to change behavioural intentions and health behaviour. In the Theory of Planned Behaviour it is suggested that intention of an individual to perform a given behaviour is the most proximal determinant of behaviour [24]. Intention, in turn, is predicted by the individual’s attitude toward the behaviour, subjective norm and the degree of perceived behavioural control (or self-efficacy) [24]. Nevertheless, prior to act upon these determinants, people must be made aware of the positive and negative consequences of a certain behaviour [25].

A theory- and evidence-based intervention was developed aiming to promote active transport for short distance travel (< eight kilometres; [26]) to various destinations among older adolescents (17–18 years). The intervention was implemented in the existing course ‘Driving Licence at School’, a project of the Flemish Foundation for Traffic Knowledge in secondary schools in Flanders (Belgium) [27]. Each year, over 40,000 secondary school students aged 17 and older have the opportunity to participate in this project [27]. Within this project, older adolescents receive free car driving theory training at school (eight hours in general and technical secondary education; ten hours in vocational secondary education) from qualified driving instructors. Thus, this existing project provided a good opportunity to reach a large group of young people at a critical stage of life regarding transport behaviour.

The present study aimed to examine the effect of the intervention on psychosocial factors including intention to use active transport after obtaining a driving licence, attitude (perceived benefits and perceived barriers), subjective norm, self-efficacy, habit and awareness towards active transport. Participants were also asked to complete process evaluation measures.

## Methods

### Study design and protocol

A matched control three-arm study was conducted and consisted of a pre-test post-test design with intervention and control schools in Flanders. A supplementary two-hour lesson promoting active transport was implemented as the last lesson in the course ‘Driving Licence at School’ in intervention schools (intervention group 1). Individuals in intervention group 2 received this active transport lesson and, in addition, they were asked to become a member of a Facebook group on active transport. Participants in the control group only attended the regular course ‘Driving Licence at School’ without the active transport lesson or the Facebook group. Qualified driving instructors gave both the regular course ‘Driving Licence at School’ and the supplementary active transport lesson as one package. Participation in the active transport lesson was obligatory for all adolescents participating in the course ‘Driving Licence at School’ in the intervention schools.

**Fig 1:**
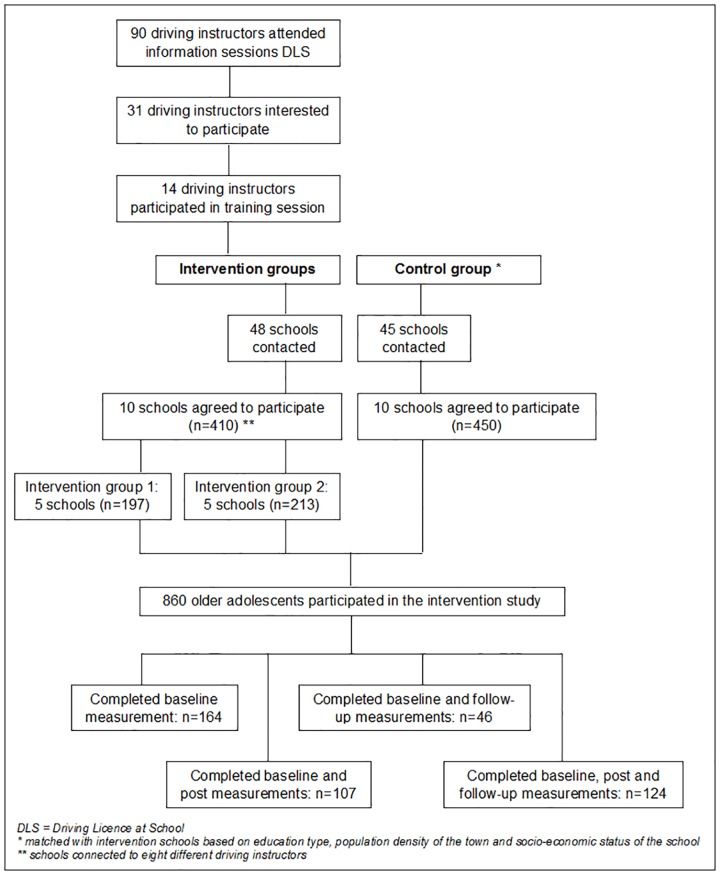
Flow chart of participant enrolment and progression through the study.

As a first step in the recruitment process, qualified driving instructors participating in the ‘Driving Licence at School’ project were recruited to teach the active transport lesson (see Fig 1). Driving instructors were recruited at annually organised information sessions of the Flemish Foundation for Traffic Knowledge on the ‘Driving Licence at School’ project. Of the ninety attending driving instructors, 31 (34%) indicated they were interested in the research project and were invited to attend a specific training session organised by the research team. This training session consisted of (a) a short introduction on the aim of the research project, (b) practicalities including instructions on recruitment of schools, planning of the active transport lesson, questionnaires and informed consents, and teaching materials and (c) a demonstration of the active transport lesson. Eventually, fourteen out of 31 invited driving instructors (45%) participated in this training session.

Consecutively, all schools (n = 48) in which these 14 instructors planned to teach the course ‘Driving Licence at School’ during the school year 2014–2015 were contacted to participate in the research project as intervention schools (see Fig 1). These schools were asked if they were willing to let their pupils attend the active transport lesson (at school; during or after school hours) and to motivate them to complete all measurements. This resulted in 10 schools (21.3%) with a total of 410 pupils attending the course ‘Driving Licence at School’ agreeing to participate in the study. Participating schools were of different educational types (general, technical and vocational secondary education) and located in both (semi-)urban and rural areas. Participating schools were connected to eight different driving instructors, the schools connected to the other six driving instructors were not willing to participate. Intervention schools, stratified by educational type, were randomly assigned to intervention group 1 (5 schools) or 2 (5 schools). After recruitment of the intervention schools, another convenience set of schools which were matched with intervention schools based on education type, population density of the town [28] and socio-economic status of the school [29] were recruited to participate as control schools. In total, 45 schools were contacted to participate in the research project as control schools. Ten schools (22.2%) with a total of 450 pupils attending the course ‘Driving Licence at School’ agreed to participate. Adolescents in the control group attended the regular course ‘Driving Licence at School’ and were asked to complete all measurements. Adolescents were eligible for participation in this study if they attended general, technical or vocational secondary education and participated in the course ‘Driving Licence at School’. A flow chart of participant enrolment and progression through the study is provided in Fig 1.

Before the start of the course ‘Driving Licence at School’, participants in the intervention and control groups completed a paper-and-pencil or an online questionnaire either at school or at home (baseline). One week after the active transport lesson, participants in both intervention groups completed the same questionnaire (post-test). Participants in the control group completed this questionnaire one week after the last lesson of ‘Driving Licence at School’. The follow-up measurement was performed two months after either the active transport lesson (intervention groups) or the last lesson of ‘Driving Licence at School’ (control group).

At the start of each questionnaire, all older adolescents were informed in writing that data would be processed anonymously and that consent was automatically obtained when they voluntarily completed the questionnaire. Since most pupils were under aged, written passive informed consent was obtained from all parents. If parents did not agree to let their child complete one or more questionnaires, a signed informed consent form had to be returned to the researchers. The study protocol was approved by the medical ethical committee of the Vrije Universiteit Brussel (January 12, 2012; B.U.N. 143201112745). The authors confirm that all ongoing and related trials for this intervention are registered. The trial was registered (NCT02823197) after starting participant recruitment since, at the start of the intervention, authors were not aware of the necessity of trial registration. The complete date range for patient recruitment and follow-up was June 1, 2014 up to September 30, 2015. The protocol for this trial and supporting TREND checklist are available as supplementary material (S1 Protocol and S1 TREND Checklist).

### Intervention

A stepwise approach was used to develop the intervention for which the Theory of Planned Behaviour was used as a theoretical backbone [24]. Designing a theory- and evidence-based intervention to promote active transport requires a comprehensive understanding of the correlates of active transport [30]. Therefore, firstly, a qualitative and quantitative study was conducted prior to the development of the intervention [31, 32]. Within these studies, factors related to active, public and passive transport among older adolescents were investigated. Based on these results, existing evidence [26, 33], and the Theory of Planned Behaviour, it was decided to focus on the following psychosocial determinants in the intervention: attitude (perceived benefits and perceived barriers), subjective norm, self-efficacy, awareness and habit towards active transport, and intention to use active transport after obtaining a driving licence. Secondly, based upon the list of theoretical methods for behaviour change published by Bartholomew et al. (2013) [25], theory-based methods were selected to influence the targeted determinants. For example, the selected theoretical method ‘consciousness raising’ targeted changes in the determinants ‘awareness’ and ‘attitude’. An overview of the methods used per determinant can be found in Table 1.

**Table 1:** Overview of the elements included in the active transport lesson and their corresponding determinants and theory-based methods used.

Element of the active transport lesson	Description	Determinant(s)	Method(s)
1) Brief introduction	The purpose of the lesson was explained and importance to always choose consciously between transport modes, even after obtaining a driving licence, was stressed.	- awareness - habit	- persuasive communication
2) Quiz	An introductory quiz was held to emphasize the importance and a advantages of physical activity and active transport.	- awareness - attitude	- persuasive communication - belief selection - consciousness raising
3) Enumeration of destinations	Participants were asked to sum up destinations they go to by foot, by bicycle and by car. They were also asked to indicate which walk- and cycle trips they would replace by car trips after obtaining a driving licence.	- awareness	- discussion
4) Enumeration and PowerPoint presentation on benefits of active transport	Participants were asked to sum up benefits of active transport, after which a PowerPoint presentation on benefits of active transport was provided.	- awareness - attitude - habit	- persuasive communication - active learning - belief selection
5) Enumeration of barriers of active transport and PowerPoint presentation on overcoming barriers of active transport	Participants were asked to sum up barriers of active transport, after which a PowerPoint presentation was given with tips and ideas on how to overcome barriers of active transport.	- awareness - attitude - habit - self-efficacy	- persuasive communication - active learning - belief selection
6) PowerPoint presentation on travelling longer distances	Alternatives to private car use are offered to travel longer distances in a sustainable way.	- awareness - attitude - habit - self-efficacy	- persuasive communication - belief selection
7) Movie on benefits of active transport	A short and amusing movie was shown in which a race through London between public transport, a car, a boat and a bicyclist is won by the bicyclist.	- awareness - attitude	- belief selection
8) Cases	Cases describing the transport behaviour of a fictitious person were given to small groups of participants which were asked to discuss how to motivate the fictitious person to choose for active transport in certain circumstances by helping him/her to overcome barriers.	- attitude - subjective norm	- discussion
9) Statements	Statements on motivation to comply with the norm of significant others were given to small groups of participants which were asked to discuss these statements.	- attitude - subjective norm	- discussion
10) Concluding message	A concluding message was given in which the importance to always choose consciously between transport modes, even after obtaining a driving licence, was stressed.	- awareness	- persuasive communication

Thirdly, the active transport lesson was developed by two researchers (HV and DS). The active transport lesson consisted of 10 elements of which an overview is provided in Table 1. Once a first draft of the lesson was completed, people from different professional domains such as researchers, policy co-operators from the Flemish Foundation for Traffic Knowledge and two qualified driving instructors were asked to provide open written (researchers) or open verbal (policy co-operators and driving instructors) feedback on the active transport lesson. Afterwards, the lesson was adapted according to their comments and remarks. In general, it was suggested to formulate the content as short and as clear as possible. Furthermore, it was decided to add some extra information after each question in the quiz (which was one part of the lesson) to provide participants with sufficient background information. Although the focus of the lesson was on short distance travel, some slides promoting public transport were added to illustrate that for longer distances public transport is a suitable transport mode with several advantages (e.g. no need to search for a parking lot).

In the next step, the second draft of the active transport lesson was used for pretesting in the target group as well as in an expert group. Two pre-tests were conducted in the target group; one in general secondary school students (n = 20; 17.6±0.6 years; 65.0% female) and one in vocational secondary school students (n = 10; 17.3±0.5 years; 66.7% female). Two additional pre-tests were conducted among expert groups, one among Master students of Physical Education and Movement Sciences (n = 5) who followed a course on health promotion and one among Public Health researchers (n = 8). These pre-test lessons were delivered by two researchers and were followed by a semi-structured group interview (see S1 Table) in which the audience was asked to provide feedback on all aspects of the lesson. The results of these semi-structured group interviews were used to adapt the lesson into a final version. The main results of the semi-structured group interviews and the corresponding adaptations made to the intervention are presented in Table 2. The final version of the active transport lesson was used for implementation. The active transport lesson consisted of 10 elements and lasted for approximately 90 minutes. An overview of these elements, and their corresponding determinants and methods used, is provided in Table 1.

**Table 2:** Main results semi-structured group interviews and corresponding adaptions.

Feedback semi-structured group interviews	Adaptations intervention
Stronger emphasis should be put on the focus of the lesson (i.e. short distance travel). *“Maybe I would emphasize more and right from the beginning (of the lesson) that it is on cycling to go somewhere for short distance travel*.*”*	In the introduction section of the intervention a few sentences were added to emphasize that the lesson was on the promotion of active transport for short distance travel. It was also explained what was meant with ‘short distances’.
Some items need more explanation or need to be rephrased in order that all participants would clearly understand everything. *“Yes*, *for some questions you have to mention ‘per year’ or…because…It was not clear*, *is the question per year or per day…”*	The wording of some parts of the lesson was slightly changed and more specified.
The section on bicycle and car sharing systems needs more detail because most adolescents have no experience with it at all. *“Concerning Cambio (car sharing system)*, *euhm… I do not know what that is*. *So maybe just explain where those Cambio-places are located*. *I have never seen it before*, *so I do not know anything about it*.*”*	Some extra information on bicycle and car sharing systems was added (e.g. extra information on the location of the systems and how the systems work).
A small group task in which adolescents have to motivate a fictitious person to walk or cycle for transport is preferred over a task in which they have to motivate a person in their class who is not motivated to walk or cycle. *“Each group receives one fictitious situation and searches for an answer…a solution*.*”*	Cases were developed describing the transport behaviour of a fictitious person which they had to motivate to walk/cycle for short distances. Afterwards a few groups had to present in front of the class how they would motivate their fictitious person.
It should be more clear whether public transport use is something that is encouraged or discouraged. *“In the beginning (of the lesson) it seemed that public transport use was discouraged*. *And at the end (of the lesson) it was promoted*. *That was a bit strange to me*. *Maybe you could solve that*.*”*	Although the lesson was on the promotion of active transport for short distance travel, it was more strongly emphasized in several parts of the lesson that public transport is a suitable transport mode which is preferred over car use when longer distances need to be travelled.

**Fig 2:**
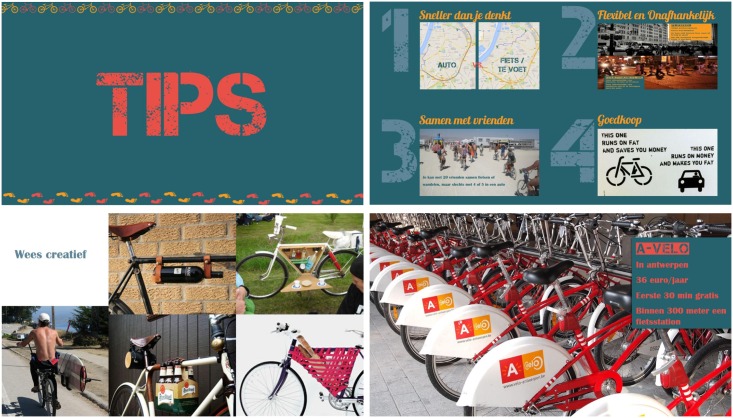
Examples of slides developed for the active transport lesson.

The active transport lesson was interactive and all elements of the lesson were made as visually appealing as possible for the older adolescents (see Fig 2) [34].

**Fig 3:**
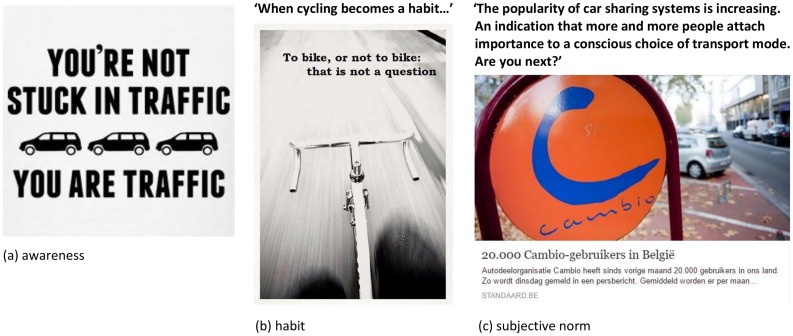
Examples of Facebook posts targeting (a) awareness; (b) habit and (c) subjective norm.

Apart from participating in the active transport lesson, individuals in intervention group 2 were asked to become a member of a Facebook group on the promotion of active transport. This Facebook group was developed in order to be able to reach participants over a longer period of time after the active transport lesson. Posts for the Facebook group were composed by a researcher (HV) and consisted of cartoons, pictures, newspaper articles, fun facts or video’s, sometimes accompanied by a message. Each post focussed on at least one of the targeted determinants. At the end of the development process, an expert group consisting of 12 Public Health researchers was asked to provide feedback on the Facebook posts. Each post was revised by three university researchers. Additionally, a member of the target group was also asked to provide feedback on the Facebook posts. The posts for the Facebook group were adapted according to the feedback. Some posts were deleted and replaced by other posts. For some posts, which initially only consisted of a cartoon or picture, an extra message was added in order to comply sufficiently with the targeted determinant. For other posts, the message was adapted. Examples of the posts are provided in Fig 3.

After the active transport lesson, a researcher (HV) posted three posts per week to the Facebook group during an eight-week period. So, participants received a total of 24 Facebook posts. During this eight-week period, four posts per determinant were launched. The Facebook group was “closed”, which means it had following restrictions: (1) Anyone could find the group and see who is in it, but only members could see posts; (2) Any member could add members, but an administrator needed to approve them; (3) Only administrators could post to the group; (4) All group posts needed to be approved by an administrator.

### Measurements

#### Effect evaluation

Socio-demographic information (i.e. gender, age, school, educational type, education father, education mother, height, weight and home address) and participants’ transport behaviour were collected at baseline using a self-reported questionnaire. Height and weight were used to calculate Body Mass Index (BMI). To assess transport behaviour, questions derived from the validated International Physical Activity Questionnaire (IPAQ) [35, 36] were used. Participants were asked to report frequency (days/week) and average daily duration of active transport (walking and cycling), public transport (train, tram, bus, metro) and passive transport (car, moped, motorcycle) within the last seven days, both to school and to other destinations. Weekly minutes per transport mode were calculated by multiplying frequency and duration of trips. Psychosocial factors such as intention to use active transport after obtaining a driving licence, attitude (perceived benefits and perceived barriers), subjective norm, self-efficacy, habit and awareness towards active transport were collected at the three time points. Questions on these psychosocial factors adhered to the guidelines described in a manual about constructing questionnaires based on the Theory of Planned Behaviour [37]. Furthermore, the questions were based on an existing questionnaire [38], and were adjusted to the specific target group according to the results of a prior explorative qualitative study [31]. A summary of these psychosocial measures is shown in Table 3.

**Table 3:** Summary of psychosocial measures and internal consistency (Cronbach α; at baseline).

Factor	Number of items	Response category	Cronbach α
Intention	3 items (e.g. how much do you want to keep using active transport for short distances after obtaining a driving licence)	five-point scale^a^	0.955
Perceived benefits	17 items (e.g. health, cost, parking lot, independence,…)	five-point scale^a^	0.911
Perceived barriers	21 items (e.g. time, accidents, weather, sweating,…)	five-point scale^b^	0.919
Subjective norm	3 items (family, friends, partner)	five-point scale^a^	0.866
Self-efficacy	11 items (e.g. bad weather, darkness, when tired,…)	five-point scale^c^	0.872
Habit	4 items (e.g. walking or cycling for transport is something I automatically do)	five-point scale^a^	0.917
Awareness	8 items (e.g. ecology, health benefits, private car ownership,…)	five-point scale^d^	0.419

^a^ five-point scale from 1 (strongly disagree) to 5 (strongly agree)

^b^ five-point scale from 1 (never) to 5 (always)

^c^ five-point scale from 1 (know I cannot do it) to 5 (know I can do it)

^d^ five-point scale from 1 (I know this is not correct) to 5 (I know this is correct)

#### Process evaluation

Participants in both intervention groups were asked to evaluate the active transport lesson at the post measurement by means of a process evaluation questionnaire. Participants who were allocated to the Facebook condition (intervention group 2) were also asked to evaluate the Facebook group at the follow-up measurement. A summary of the measures on the evaluation of the active transport lesson and the Facebook group is shown in Table 4.

**Table 4:** Summary of the process evaluation measures and descriptives.

	Response category	Response alternatives	Mean (SD), %
**Active transport lesson**			
If you could choose, would you have followed the lesson voluntarily?		Yes, I am interested in the topic;	37.5
		No, but eventually it was interesting;	31.3
		No, I am not interested in the topic	31.3
Attractiveness content	4 items; five-point scale^a^	e.g.: How much do you agree that the lesson was useful?	3.5 (1.1)
Adapted to target group	1 item; five-point scale^a^	How much do you agree that the lesson was adapted to your age group?	3.7 (1.0)
Difficulty content	1 item; five-point scale^a^	How much do you agree that the lesson was difficult?	2.1 (1.1)
Was the lesson able to motivate you to use active transport?		I was already motivated before;	71.5
Which are the reasons you were less motivated before?		Yes, I was less motivated before;	10.9
		I am aware now that it is better for my health, the environment,…;	39.1
		I am more aware of the benefits;	43.5
		I know better how to cope with the disadvantages now;	13.0
		I learned new things which can help me to choose the right travel mode	4.3
Which are the reasons you are still not motivated?		No, I am still not motivated	17.6
		The lesson was no encouragement for me;	27.8
		I was not interested;	13.9
		I think it is not necessary to walk or cycle more;	33.3
		I find it difficult to really do this	16.7
**Facebook group**			
Did you join the Facebook group?		Yes, I joined the Facebook group;	32.8
		No, I did not join the Facebook group	67.2
Why did you not join the Facebook group?		I do not have a Facebook account;	27.3
		I forgot to join the Facebook group;	30.3
		I did not want to received messages regarding the topic;	27.3
		I did not want the researchers to see my Facebook profile	15.2
Attractiveness Facebook posts	5 items; five-point scale^a^	e.g. How much do you agree that the Facebook posts were useful?	3.0 (1.0)
Adapted to target group	1 item; five-point scale^a^	How much do you agree that the Facebook group was adapted to your age group?	3.3 (1.1)
Was the Facebook group able to motivate? you to use active transport?		I was already motivated before;	60.0
		Yes, I was less motivated before;	30.0
		No, I am still not motivated	10.0

^a^ five-point scale from 1 (strongly disagree) to 5 (strongly agree)

### Data analyses

Data were analysed using IBM SPSS Statistics version 22 (see S1 Dataset Effect Evaluation and S1 Dataset Process Evaluation). To check for differences between the control group, intervention group 1 and intervention group 2 at baseline, one-way ANOVA, Kruskal-Wallis and Chi-Square tests were conducted. Linear mixed models analyses were performed to assess the effectiveness of the intervention on psychosocial determinants (dependent variables). The model included three hierarchically ordered levels: school, participant and time. Intercepts were allowed to vary randomly at the school and participant level, all slopes were assumed to be fixed. Linear mixed models analyses allowed us to include all available measurements, even if participants completed only one or two measurements. Mixed models have advantages over fixed effects models in the treatment of missing values of the dependent variable. Mixed models are capable of handling the imbalance caused by missing observations and yield valid inferences if the missing observations are missing at random [39]. In addition, linear mixed models can handle correlated data such as responses of students from the same school. The eight items representing awareness were included separately due to low internal consistency (Cronbach’s alpha<0.6). Since only a small sample (n = 20) of older adolescents in intervention group 2 actually became a member of the Facebook group, an extra set of linear mixed models analyses were performed to identify differences in intervention effects between those who became a member of the Facebook group and those who did not (from baseline to follow-up). P-values < 0.05 were considered statistically significant and p-values between 0.05 and 0.10 were considered borderline significant. Descriptive statistics were calculated to analyse the process evaluation measures.

## Results

### Effect evaluation

In total, 441 older adolescents with at least a baseline measurement (51.3% on a total of 860 adolescents participating in ‘Driving Licence at School’ in both intervention and control schools) were included in the study. Of these individuals, 124 participants completed baseline, post and follow-up measurements, 107 participants completed baseline and post measurements, 46 participants completed baseline and follow-up measurements, and 164 participants only completed the baseline measurement. General characteristics of the study population at baseline are shown in Table 5. Of the total sample, 56.8% was female and mean age was 17.4 (0.7) years. Significantly more girls were represented in intervention group 1 compared to the control group, and intervention group 1 had a significantly lower BMI compared to intervention group 2 and the control group. Furthermore, significantly more participants with a lower socio-economic status (SES) were represented in intervention group 2 compared to intervention group 1 and the control group. In the control group, significantly more participants from general and technical secondary education were represented compared to intervention group 2. Therefore, all analyses were adjusted for gender, BMI, SES and educational type. Furthermore, the analyses were also adjusted for season as not all measurements were taken at the same time for the different schools.

**Table 5:** Characteristics of both the intervention groups and the control group at baseline (%, Mean (SD)).

	Intervention group 1 ^a^	Intervention group 2 ^b^	Control group ^c^	F-value or Chi^2^-value
	n = 132	n = 163	n = 146	
Gender (% female)	65.2 °	55.6	50.7 ^£^	6.080**
Age (yrs)	17.4 (0.7)	17.5 (0.7)	17.3 (0.6)	4.132
BMI (kg/m^2^)	21.1 (2.7) ^§^^,^°	22.2 (3.6) ^£^	22.5 (3.8) ^£^	7.568**
Socio-economic status (% low SES)^1^	40.9 ^§^	60.2 ^£^^,^°	43.0 ^§^	9.022**
General/technical studies (%)	58.0	50.3 °	66.4 ^§^	8.225**
Distance home-school (km)	5.7 (4.9)	6.2 (6.1)	6.4 (7.8)	0.354
Active transport (minutes/week)	221.5 (205.7)	183.4 (279.0)	207.9 (356.2)	0.658
Public transport (minutes/week)	193.2 (309.3)	239.4 (272.7)	236.5 (421.3)	0.795
Passive transport (minutes/week)	117.6 (167.5)	130.6 (170.3)	128.2 (278.7)	0.146

*p<0.10,

**p<0.05,

***p<0.001.

^£^ significant difference with intervention group 1;

^§^ significant difference with intervention group 2;

° significant difference with control group.

^a^ Only active transport lesson;

^b^ Active transport lesson and Facebook group;

^c^ Neither active transport lesson nor Facebook group.

^1^ Low SES (% no parent has a Bachelor’s degree or higher)—high SES (% at least one parent has a Bachelor’s degree or higher).

**Fig 4:**
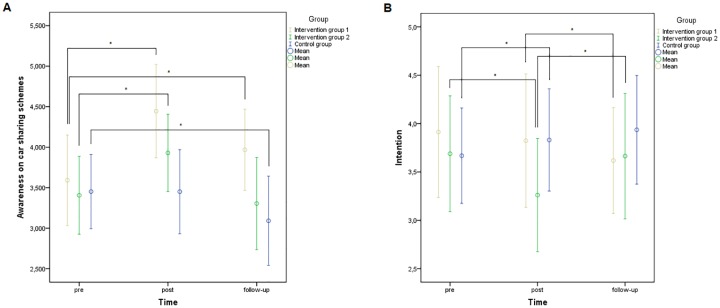
Evolution of psychosocial variables ‘awareness on car sharing schemes’ and ‘intention to use active transport after obtaining a driving licence’ according to group and time.

Average item scores for the psychosocial variables according to group and time, and results obtained from the linear mixed models analyses are shown in Table 6. Only for awareness regarding the existence of car sharing schemes and intention to use active transport after obtaining a driving licence, significant intervention effects were found (p = 0.009 and p = 0.014, respectively). For awareness regarding the existence of car sharing schemes, significant interaction effects were found with an increase in awareness from baseline to post measurement within intervention group 1 (p = 0.001) and intervention group 2 (p = 0.030) compared to the control group in which no change was found (see Fig 4(A)). Finally, a significant interaction effect (p = 0.043) was found with an increase in awareness from baseline to follow-up measurement within intervention group 1 and a decrease in awareness from baseline to follow-up measurement within the control group (see Fig 4(A)). Regarding intention to use active transport after obtaining a driving licence, a significant interaction effect (p = 0.031) was found with an increase in intention from post to follow-up measurement within intervention group 2 compared to intervention group 1 in which a slight decrease was found (see Fig 4(B)). However, from baseline to post measurement, a significant decrease in intention was found within intervention group 2 compared to the control group in which a slight increase was found (see Fig 4(B)).

**Table 6:** Average item scores and time and interaction effects for psychosocial variables in the total sample.

		Pre	Post	Follow-up	Time	Time*group
		*mean (CI*^£^*)*	*mean (CI)*	*mean (CI)*	*p-value*	*p-value*
Intention ^a^	IG 1	3.9 (3.2; 4.6)	3.8 (3.1; 4.5)	3.6 (3.1; 4.2)	0.321	**0.014**
	IG 2	3.7 (3.1; 4.3)	3.3 (2.7; 3.8)	3.7 (3.0; 4.3)		
	CG	3.7 (3.2; 4.2)	3.8 (3.3; 4.4)	3.9 (3.4; 4.5)		
Perceived benefits ^a^	IG 1	3.8 (3.4; 4.3)	3.9 (3.5; 4.3)	3.7 (3.4; 4.0)	0.435	0.301
	IG 2	3.7 (3.3; 4.0)	3.6 (3.3; 4.0)	3.6 (3.2; 4.0)		
	CG	3.7 (3.4; 4.1)	3.9 (3.6; 4.3)	3.9 (3.5; 4.2)		
Perceived barriers ^b^	IG 1	2.1 (1.6; 2.5)	2.0 (1.5; 2.4)	2.2 (1.8; 2.6)	0.328	0.229
	IG 2	2.3 (1.9; 2.7)	2.5 (2.1; 2.9)	2.5 (2.1; 3.0)		
	CG	2.1 (1.8; 2.5)	2.1 (1.8; 2.5)	2.4 (2.0; 2.8)		
Subjective norm ^a^	IG 1	2.3 (1.8; 2.9)	2.1 (1.5; 2.7)	2.2 (1.7; 2.7)	0.389	0.372
	IG 2	2.6 (2.0; 3.0)	2.7 (2.2; 3.2)	2.5 (1.9; 3.0)		
	CG	2.6 (2.1; 3.1)	2.4 (1.8; 2.9)	2.4 (1.8; 3.0)		
Self-efficacy ^c^	IG 1	3.0 (2.6; 3.5)	3.2 (2.7; 3.7)	3.0 (2.6; 3.4)	0.205	0.566
	IG 2	2.9 (2.5; 3.3)	2.9 (2.5; 3.3)	2.9 (2.5; 3.4)		
	CG	3.1 (2.7; 3.4)	3.3 (2.9; 3.6)	3.2 (2.8; 3.6)		
Habit ^a^	IG 1	3.7 (2.9; 4.4)	3.8 (3.0; 4.6)	3.8 (3.2; 4.5)	0.114	0.551
	IG 2	3.3 (2.6; 4.0)	3.3 (2.6; 4.0)	3.7 (3.0; 4.5)		
	CG	3.5 (2.9; 4.1)	3.8 (3.1; 4.4)	4.1 (3.5; 4.8)		
Awareness on ecology ^d^	IG 1	4.0 (3.5; 4.5)	3.9 (3.4; 4.5)	3.7 (3.3; 4.2)	0.108	0.252
	IG 2	4.0 (3.5; 4.4)	3.6 (3.1; 4.0)	3.6 (3.1; 4.1)		
	CG	4.1 (3.7; 4.5)	4.0 (3.6; 4.5)	4.1 (3.6; 4.6)		
Awareness on travel speed ^e^	IG 1	2.8 (1.8; 3.7)	3.4 (2.4; 4.4)	3.4 (2.6; 4.2)	**<0.001**	0.180
	IG 2	2.4 (1.5; 3.3)	3.2 (2.3; 4.1)	3.3 (2.3; 4.3)		
	CG	3.0 (2.2; 3.7)	3.1 (2.3; 3.8)	3.5 (2.7; 4.3)		
Awareness on physical activity ^f^	IG 1	4.2 (3.6; 4.7)	4.3 (3.7; 4.9)	3.9 (3.4; 4.5)	0.331	0.309
	IG 2	4.0 (3.5; 4.5)	3.6 (3.1; 4.1)	3.7 (3.1; 4.3)		
	CG	4.0 (3.5; 4.4)	4.0 (3.4; 4.5)	3.6 (3.1; 4.2)		
Awareness on health benefits ^g^	IG 1	3.4 (2.6; 4.2)	3.6 (2.8; 4.4)	3.3 (2.6; 4.0)	0.932	0.267
	IG 2	3.2 (2.6; 3.9)	2.9 (2.2; 3.5)	3.2 (2.5; 4.0)		
	CG	3.4 (2.8; 4.0)	3.5 (2.8; 4.1)	3.3 (2.6; 4.1)		
Awareness on private car ownership ^h^	IG 1	3.3 (2.5; 4.1)	3.3 (2.6; 4.1)	3.4 (2.8; 4.1)	0.660	0.415
	IG 2	3.1 (2.4; 3.7)	3.0 (2.4; 3.6)	2.9 (2.2; 3.6)		
	CG	3.5 (3.0; 4.1)	3.4 (2.7; 4.0)	2.9 (2.3; 3.6)		
Awareness on public transport use ^i^	IG 1	3.9 (3.4; 4.4)	3.9 (3.4; 4.5)	3.8 (3.3; 4.3)	0.485	0.659
	IG 2	3.7 (3.2; 4.2)	3.5 (3.0; 3.9)	3.4 (2.8; 3.9)		
	CG	3.8 (3.3; 4.2)	3.9 (3.4; 4.4)	3.5 (3.0; 4.1)		
Awareness on bicycle sharing schemes ^j^	IG 1	4.2 (3.6; 4.7)	4.5 (3.9; 5.1)	4.0 (3.5; 4.5)	**0.050**	0.185
	IG 2	4.0 (3.5; 4.5)	3.8 (3.3; 4.3)	3.2 (2.7; 3.8)		
	CG	3.9 (3.4; 4.3)	3.9 (3.4; 4.4)	3.5 (3.0; 4.0)		
Awareness on car sharing schemes ^k^	IG 1	3.6 (3.0; 4.2)	4.4 (3.9; 5.0)	4.0 (3.5; 4.5)	**<0.001**	**0.009**
	IG 2	3.4 (2.9; 3.9)	3.9 (3.5; 4.4)	3.3 (2.7; 3.9)		
	CG	3.5 (3.0; 3.9)	3.5 (2.9; 4.0)	3.1 (2.5; 3.6)		

IG 1 = intervention group 1 (only active transport lesson); IG 2 = intervention group 2 (active transport lesson and Facebook group); CG = control group (neither active transport lesson or Facebook group);

^*£*^ 95% confidence interval (CI);

^a^ five-point scale from 1 (strongly disagree) to 5 (strongly agree);

^b^ five-point scale from 1 (never) to 5 (always);

^c^ five-point scale from 1 (know I cannot do it) to 5 (know I can do it);

^d^ ‘using active transport is beneficial to the environment’;

^e^ ‘using active transport is not always slower compared to using a car’;

^f^ ‘using active transport contributes to sufficient physical activity’;

^g^ ‘using active transport regularly has a positive influence on my health’;

^h^ ‘owning a private car is necessary’;

^i^ ‘for longer distances public transport combined with active transport is an acceptable alternative’;

^j^ ‘there are systems which make it possible to rent a bicycle when needed’;

^k^ ‘there are systems which make it possible to rent a car when needed’; questions on awareness: five-point scale from 1 (I know this is not correct) to 5 (I know this is correct).

An extra set of mixed models analyses was not able to detect differences in intervention effects from baseline to follow-up measurement between participants in intervention group 2 who joined the Facebook group and participants who did not join the Facebook group.

### Process evaluation

Results of the process evaluations are shown in Table 3. In total, 170 out of 295 older adolescents allocated to one of both intervention groups (57.6%) completed the process evaluation measures on the active transport lesson. About one-third (37.5%) of participants indicated that they would have participated voluntarily in the active transport lesson if they were not obliged to. Furthermore, 31.3% of participants indicated that they would not have participated voluntarily but, eventually, they thought that the active transport lesson was interesting. The same percentage of older adolescents indicated that they were not interested in the topic. The older adolescents reported that the content of the active transport lesson was fairly attractive (3.5 (1.1); five-point scale) and adapted to the target group (3.7 (1.0); five-point scale). The content of the lesson was rated as not difficult (2.1 (1.1); five-point scale). Furthermore, the manner of teaching by the driving instructor was rated good (4.0 (0.8); five-point scale). A large part of the sample (71.5%) indicated that they were already motivated to walk or cycle for short distance travel before the active transport lesson. Furthermore, 10.9% indicated that they were less motivated before the active transport lesson compared to after the active transport lesson, of which 39.1% indicated that they are aware now that walking and cycling is better for their health, the environment,…. Nearly half of the sample (43.5%) indicated that they are more aware of the benefits of walking and cycling, whereas 13.0% indicated they know how to cope with the disadvantages of walking and cycling better now. Finally, 4.3% indicated they learned new things which can help to choose the right travel mode. Nevertheless, 17.6% of participants reported that they were still not motivated to walk or cycle for short distance travel after the active transport lesson. Of these participants, 27.8% indicated that the active transport lesson was no encouragement for them and 13.9% said they were not interested in the lesson. Approximately one third of the sample (33.3%) indicated that they think it is not necessary to walk or cycle more and 16.7% indicated that it is difficult to really do this.

A total of 61 out of 163 older adolescents allocated to intervention group 2 (37.4%) completed the process evaluation on the Facebook group. Approximately one third of participants in intervention group 2 (32.8%) indicated that they joined the Facebook group. Of those who did not join the Facebook group, 27.3% reported that they do not have a Facebook account, 30.3% reported that they forgot to join when they arrived at home (these participants did not own a smartphone and were not able to join the Facebook group during the active transport lesson). Furthermore, 27.3% did not want to receive messages regarding the topic and 15.2% did not want the researchers to see their Facebook profile. Those who joined the Facebook group, thought that the posts were sometimes interesting/sometimes not interesting (3.0 (1.0)); five-point scale). Furthermore, they indicated that the posts were adapted to the target group (3.3 (1.1)); five-point scale). In total, 60.0% of older adolescents indicated that they were already motivated to walk or cycle for short distance travel before they joined the Facebook group, 30.0% indicated that they were less motivated before they joined and 10.0% indicated they were still not motivated to walk or cycle for short distance travel.

## Discussion

Although the developed intervention was theory- and evidence-based, the main finding of this study was that implementing an extra two-hour lesson on the promotion of active transport within the eight-hour course ‘Driving Licence at School’ was, in general, not effective in changing psychosocial factors related to active transport. The addition of a Facebook group on active transport was also not sufficient to change psychosocial factors.

Although the process evaluation revealed that the intervention was evaluated as fairly attractive and adapted to the target group, it was not able to induce change. However, the presence of ceiling effects has to be taken into account since 71.5% of participants indicated they were already motivated to use active transport for short distance travel before the active transport lesson. Several previous interventions promoting active transport targeted those who were motivated to change their behaviour in favour of active transport modes [16, 20]. However, it is important to reach those who are less motivated to walk or cycle for transport too. By integrating the intervention into the project ‘Driving Licence at School’, adolescents from participating schools were obliged to follow the active transport lesson. Since these older adolescents were participating in a project in which they received car driving theory training, they were probably motivated to learn to drive a car. Thus, both motivated as well as non-motivated adolescents were included in the intervention. Yet, it should be noted that it is possible that mainly adolescents who were already motivated to use active transport completed the questionnaires.

A more intensive approach possibly could have resulted in the desired intervention effects. However, implementing more lessons promoting active transport in ‘Driving Licence at School’ was not possible due to time constraints at secondary schools. Furthermore, some driving instructors mentioned that it was difficult to motivate the older adolescents to participate actively during the active transport lesson. Participating schools indicated that it would be more manageable for them to integrate (parts of) the active transport lesson into a project day. This could be a great opportunity to extend the active transport lesson with other components such as more practice-based components. By adding one or more practice-based components, older adolescents may perceive an intervention promoting active transport less intrusive and more attractive. In a school-based intervention targeting sleep problems, adolescents indicated that they preferred interactive learning opportunities such as hands-on class activities to transfer knowledge into practice [40]. Previous intervention studies targeting active travel, although among other age groups, showed that multifaceted interventions were able to increase active transport levels [21, 41]. These interventions consisted for example of information provision, cycling training, cycle repair and personalised travel planning.

Although it is essential to intervene in this age group as they are at a critical stage of life regarding transport behaviour [15], older adolescents may not be the most receptive age group for an intervention promoting active transport. Older adolescents finally get the chance to drive a car and increase their level of independent travel. Therefore, it was expected that participants’ intention to use active transport after obtaining a driving licence would decrease at post and follow-up measurement. The intervention intended to minimize this decrease as much as possible. Although integrating the active transport lesson into the course ‘Driving Licence at School’ seemed a great opportunity, this may not be the most effective approach. In addition, a study among 10–17 year olds showed that adolescents have a clear preference for non-intrusive intervention strategies over more intrusive strategies [42]. Since adolescents in intervention schools were obliged to follow the active transport lesson, it is likely that they perceived the intervention as too intrusive.

The use of social media tools (such as Facebook) for health promotion programs was perceived as a promising strategy since it is a cost-efficient method to reach large audiences and adolescents in particular [43]. A recent study among Flemish (Belgian) 12–18 year olds indicated that 89.9% of these adolescents has an active Facebook account and 86.2% log in to their account daily [44]. However, the Facebook group only had a positive effect on intention to use active transport after obtaining a driving licence. It should be noted that the low participation rate in the present study makes it difficult to draw conclusions regarding the effect of the addition of a Facebook group. The present study showed that older adolescents were suspicious to join a Facebook group developed by researchers and which had some link with their school. This phenomenon was also posited by Cobb et al. [45]. Therefore, a crucial step for future intervention studies using social media which target (older) adolescents will be to search for strategies that can convince this target group to join, for example, a Facebook group developed by researchers. Another strategy could be to involve the target group in the development of the Facebook group and to let them compose potential Facebook posts. This will probably also result in more interaction between the members of the Facebook group. Wójcicki et al. [46] also suggested that a more participatory approach might benefit active engagement in a social media intervention among adolescents.

In accordance with other intervention studies among adolescents reporting high drop-out rates [47, 48], the present intervention study showed that older adolescents are a difficult age group to target. Although the active transport lesson was organised at school and the older adolescents had the opportunity to complete the measurements at school, there was a certain level of resistance and apathy towards the study. It was very difficult to convince the older adolescents to complete all measurements which is reflected by the large drop-out in this study. Future intervention studies among adolescents might benefit from eliminating redundant questions and keep questionnaires/measurements as short as possible without missing necessary information [49].

### Limitations and strengths

A first limitation of this study is that for those psychosocial factors for which a significant intervention effect was found, the effect sizes appeared to be very small, especially for intention to use active transport after obtaining a driving licence. Therefore, these results need to be interpreted with caution. Second, for those few variables for which a significant intervention effect was found, a type one error may have occurred due to multiple testing. Third, there is no long-term follow-up measurement. Although an extra follow-up measurement would have made the design stronger, it is unlikely that the adolescents would have been prepared to complete more questionnaires given the large drop-out in the short term. Fourth, self-reported questionnaires were used that may lead to social desirability bias and errors in self-observation. Fifth, only 20 adolescents joined the Facebook group which makes it difficult to draw conclusions regarding the effectiveness of the Facebook posts. Sixth, 71.5% of participants indicated they were already motivated to use active transport for short distances before the lesson which could have led to ceiling effects. In addition, probably the most motivated adolescents were also the ones who completed the questionnaires which may have led to biased results. Seventh, participants in vocational education in the last two years of secondary school were over-represented in the present sample compared to the total population of adolescents in Flanders during the school year 2014–2015 (42.0% versus 29.5%) [50]. Eighth, older adolescents are a specific age group which makes it difficult to generalize results to other age groups. Finally, another limitation is the large drop-out. Mixed models analyses were used to overcome this limitation.

A first strength is that the developed intervention was theory- and evidence-based, and that the intervention was developed in collaboration with policy co-operators from the Flemish Foundation for Traffic Knowledge and people in the field (e.g. driving instructors). A second strength was that the intervention was integrated in an existing course supported by the Flemish Foundation for Traffic Knowledge which annually reaches a large group of young people at a critical stage of life regarding transport behaviour. If the intervention would have been effective, this would have been a great opportunity for long term implementation.

## Conclusions

Overall, the present intervention study was not effective in changing psychosocial correlates of active transport. A lot of effort was put into motivating the older adolescents to participate actively in the intervention and to complete all measurements, yet a lot of obstacles were experienced. Future intervention studies should search for alternative strategies to motivate and involve this hard to reach target group.

## Supporting Information

S1 Dataset Effect Evaluation Raw data obtained from the questionnaires. (XLSX)

S1 Dataset Process Evaluation Raw data obtained from the questionnaires. (XLS)

S1 Protocol Trial protocol. (PDF)

S1 Table Semi-structured interview used for pre-testing. (DOCX)

S1 TREND Checklist TREND checklist. (PDF)
