# Supplementary material for: Promoting Active Transport in Older Adolescents Before They Obtain Their Driving Licence: A Matched Control Intervention Study
Source: PLoS One. 2016 Dec 29;11(12):e0168594. doi: 10.1371/journal.pone.0168594 (PMC5199110; doi:10.1371/journal.pone.0168594)
Supplement: S1 Protocol — (PDF) [file pone.0168594.s003.pdf]

More and safer mobility: testing of a conceptual model for explaining active and safe transport among adolescents and young adults

Table of contents

Background 2
Intervention 2
Objectives and research questions 2
Methods 3
Population 3
Recruitment 3
Design 3
Measurements 3
Informed consent 4

Background

Active transport has great potential to increase physical activity in adolescents and young adults since it can be easily integrated into the daily routine. However, once adolescents reach driving age their active transport levels decrease dramatically. Several previous interventions to promote walking and cycling as a mode of transport have been executed, but none of these studies focused on older adolescents (17-18 years). However, because of the possibility to obtain a driving licence in the near future, older adolescents are a at critical stage of life regarding transport behaviour. Therefore, it might be important to promote active transport at the age of 17-18 years (older adolescence), just before habitual car driving patterns get established.

Intervention

The intervention will consist of a 2-hour lesson promoting active transport which will be implemented in the course 'Driving Licence at School'. This course is a project of the Flemish Foundation for Traffic Knowledge in which secondary school students (aged 17 and older) receive 8 hours of car driving theory training at school from a qualified driving instructor. Furthermore, a Facebook group on active transport will be introduced in a subsample of older adolescents after the active transport lesson.

Objectives and research questions

The intervention aims to influence psychosocial factors of active transport since this is the first step in order to achieve a change in behaviour. The objective of the study is to examine the effect of the intervention on psychosocial factors including intention to use active transport after obtaining a driving licence, attitude (perceived benefits and perceived barriers), subjective norm, self-efficacy, habit and awareness towards active transport.

<u>Research questions</u>

- What are the effects of the intervention on following psychosocial factors: intention to use active transport after obtaining a driving licence, attitude (perceived benefits and perceived barriers), subjective norm, self-efficacy, habit and awareness towards active transport?
- How is the intervention perceived by the target group?

Methods

Population

The study sample consists of Flemish (Belgian) adolescents in the last two years of secondary school participating in the project 'Driving Licence at School'. Adolescents younger than 16 years and older than 19 years will be excluded. Adolescents are eligible for participation in the study when they are able to complete a Dutch (online) questionnaire.

Recruitment

As a first step in the recruitment process, qualified driving instructors participating in the 'Driving Licence at School' project will be recruited to teach the active transport lesson. Consecutively, schools in which these driving instructors plan to teach the course 'Driving Licence at School' during the school year 2014-2015 will be contacted to participate in the research project as intervention school. Next, control schools which will be recruited will be matched with intervention schools based on education type, population density of the town and socio-economic status of the school. At participating schools, all adolescents participating in the course 'Driving Licence at School' will receive the supplementary active transport lesson.

Design

A matched control intervention study with three arms will be conducted. Intervention group 1 will receive the supplementary active transport lesson as the last lesson in the course 'Driving License at School'; intervention group 2 also receives the active transport lesson and, in addition, they will be asked to become a member of the Facebook group on active transport. Participants in the control group will only attend the regular course 'Driving Licence at School' without the active transport lesson or the Facebook group.

Measurements

Paper-and-pencil or online questionnaires will be completed before the start of the course 'Driving License at School' (baseline), one week after the last lesson (post) and 8 weeks after the last lesson (follow-up). Socio-demographic information (i.e. gender, age, school, educational type, education father, education mother, height, weight and home address) and participants' transport behaviour will be collected at baseline. Psychosocial factors will be collected at the three time points.

Participants in both intervention groups will be asked to evaluate the active transport lesson at the post measurement by means of a process evaluation questionnaire. Participants who are allocated to the Facebook condition (intervention group 2) will be asked to evaluate the Facebook group at the follow-up measurement.

Informed consent

At the start of each questionnaire, participants will be informed that data will be processed anonymously. Furthermore, they will be informed that consent is automatically obtained when they voluntarily complete the questionnaire. Passive informed consent will be obtained from parents.
