# Supplementary material for: Promoting Active Transport in Older Adolescents Before They Obtain Their Driving Licence: A Matched Control Intervention Study
Source: PLoS One. 2016 Dec 29;11(12):e0168594. doi: 10.1371/journal.pone.0168594 (PMC5199110; doi:10.1371/journal.pone.0168594)
Supplement: S1 Table — (DOCX) [file pone.0168594.s004.docx]

S2 Table: Semi-structured interview used for pre-testing

| **Element of the lesson** | **Questions** |
| --- | --- |
| Quiz | - What did you think of   1. the design and layout of the slides?   2. the concept of the quiz?   3. the quiz questions?   4. the explanations given with the answers to the questions? - Do you have any other remarks or comments on the quiz? |
| Enumeration of destinations | - What did you think of summing up destinations you go to by foot, by bicycle and by car? |
| Enumeration and PowerPoint presentation on benefits of active transport | - What did you think of   1. the design and layout of the slides?   2. the content of the slides?   3. the explanations given with the slides? - Do you have any other remarks or comments on the PowerPoint presentation? |
| Enumeration of barriers of active transport and PowerPoint presentation on overcoming barriers of active transport | - What did you think of the enumeration of barriers of walking and cycling? - What did you think of   1. the design and layout of the slides?   2. the content of the slides?   3. the explanations given with the slides? - Do you have any other remarks or comments on the PowerPoint presentation? |
| Movie on benefits of active transport | - Did you found the sound and image of the movie clear? - Did you understand the movie? - How did you like the movie? - Did you think the movie was appropriate for this lesson? - Do you have any other remarks or comments on the movie? |
| Cases | - What did you think of   1. the assignment?   2. discussing in small groups? - Do you have any other remarks or comments on the cases? |
| Concluding message | - What did you think about the concluding message? |
| In general | - What did you think of the duration of the lesson? - What did you think of the duration of the separate elements of the lesson? - What did you think of the sequence of all the elements in the lesson? - What did you think of the teaching method? |
